# Supplementary material for: Prevalence and virulence genes of Staphylococcus aureus from food contact surfaces in Thai restaurants
Source: PeerJ. 2023 Aug 14;11:e15824. doi: 10.7717/peerj.15824 (PMC10434075; doi:10.7717/peerj.15824)
Supplement: Supplemental Information 2 [file peerj-11-15824-s002.docx]

**Crosstabs**

| **Case Processing Summary** | | | | | | |
| --- | --- | --- | --- | --- | --- | --- |
|  | Cases | | | | | |
|  | Valid | | Missing | | Total | |
|  | N | Percent | N | Percent | N | Percent |
| ica * fnbA | 200 | 100.0% | 0 | 0.0% | 200 | 100.0% |
| ica * cna | 200 | 100.0% | 0 | 0.0% | 200 | 100.0% |
| ica * bap | 200 | 100.0% | 0 | 0.0% | 200 | 100.0% |

**ica * fnbA**

| **Crosstab** | | | | | | | | | | |  |  |  |  |  |  |  |  |  |  |  |  |
| --- | --- | --- | --- | --- | --- | --- | --- | --- | --- | --- | --- | --- | --- | --- | --- | --- | --- | --- | --- | --- | --- | --- |
| Count | | | | | | | | | | |  |  |  |  |  |  |  |  |  |  |  |  |
|  | | fnbA | | | | | Total | | | |  |  |  |  |  |  |  |  |  |  |  |  |
|  |  | not found | | | found | |  |  |  |  |  |  |  |  |  |  |  |  |  |  |  |  |
| ica | not found | 158 | | | 16 | | 174 | | | |  |  |  |  |  |  |  |  |  |  |  |  |
|  | found | 13 | | | 13 | | 26 | | | |  |  |  |  |  |  |  |  |  |  |  |  |
| Total | | 171 | | | 29 | | 200 | | | |  |  |  |  |  |  |  |  |  |  |  |  |
| **Chi-Square Tests** | | | | | | | | | | | | | | | | | | | |  |  |  |
|  | | | | Value | | df | | | | Asymp. Sig. (2-sided) | | | Exact Sig. (2-sided) | | | | Exact Sig. (1-sided) | | |  |  |  |
| Pearson Chi-Square | | | | 30.379^a^ | | 1 | | | | .000 | | |  | | | |  | | |  |  |  |
| Continuity Correction^b^ | | | | 27.177 | | 1 | | | | .000 | | |  | | | |  | | |  |  |  |
| Likelihood Ratio | | | | 22.683 | | 1 | | | | .000 | | |  | | | |  | | |  |  |  |
| Fisher's Exact Test | | | |  | |  | | | |  | | | .000 | | | | .000 | | |  |  |  |
| Linear-by-Linear Association | | | | 30.227 | | 1 | | | | .000 | | |  | | | |  | | |  |  |  |
| N of Valid Cases | | | | 200 | |  | | | |  | | |  | | | |  | | |  |  |  |
| a. 1 cells (25.0%) have expected count less than 5. The minimum expected count is 3.77. | | | | | | | | | | | | | | | | | | | |  |  |  |
| b. Computed only for a 2x2 table | | | | | | | | | | | | | | | | | | | |  |  |  |
| **Directional Measures** | | | | | | | | | | | | | | | | | | | | | | |
|  | | | | | | | | | | | | | | Value | | Asymp. Std. Error^a^ | | | Approx. T | | | Approx. Sig. |
| Nominal by Nominal | | | Lambda | | | | | | Symmetric | | | | | .000 | | .000 | | | .^b^ | | | .^b^ |
|  |  |  |  |  |  |  |  |  | ica Dependent | | | | | .000 | | .000 | | | .^b^ | | | .^b^ |
|  |  |  |  |  |  |  |  |  | fnbA Dependent | | | | | .000 | | .000 | | | .^b^ | | | .^b^ |
|  |  |  | Goodman and Kruskal tau | | | | | | ica Dependent | | | | | .152 | | .071 | | |  | | | .000^c^ |
|  |  |  |  |  |  |  |  |  | fnbA Dependent | | | | | .152 | | .070 | | |  | | | .000^c^ |
| a. Not assuming the null hypothesis. | | | | | | | | | | | | | | | | | | | | | | |
| b. Cannot be computed because the asymptotic standard error equals zero. | | | | | | | | | | | | | | | | | | | | | | |
| c. Based on chi-square approximation | | | | | | | | | | | | | | | | | | | | | | |
| **Symmetric Measures** | | | | | | | | | | | | | | | | | | | | |  |  |
|  | | | | | | | | Value | | | | Asymp. Std. Error^a^ | | | Approx. T^b^ | | | Approx. Sig. | | |  |  |
| Nominal by Nominal | | | Phi | | | | | .390 | | | |  | | |  | | | .000 | | |  |  |
|  |  |  | Cramer's V | | | | | .390 | | | |  | | |  | | | .000 | | |  |  |
|  |  |  | Contingency Coefficient | | | | | .363 | | | |  | | |  | | | .000 | | |  |  |
| Interval by Interval | | | Pearson's R | | | | | .390 | | | | .092 | | | 5.955 | | | .000^c^ | | |  |  |
| Ordinal by Ordinal | | | Spearman Correlation | | | | | .390 | | | | .092 | | | 5.955 | | | .000^c^ | | |  |  |
| N of Valid Cases | | | | | | | | 200 | | | |  | | |  | | |  | | |  |  |
| a. Not assuming the null hypothesis. | | | | | | | | | | | | | | | | | | | | |  |  |
| b. Using the asymptotic standard error assuming the null hypothesis. | | | | | | | | | | | | | | | | | | | | |  |  |
| c. Based on normal approximation. | | | | | | | | | | | | | | | | | | | | |  |  |

**ica * cna**

| **Crosstab** | | | | | | | | | | |  |  |  |  |  |  |  |  |  |  |  |  |  |
| --- | --- | --- | --- | --- | --- | --- | --- | --- | --- | --- | --- | --- | --- | --- | --- | --- | --- | --- | --- | --- | --- | --- | --- |
| Count | | | | | | | | | | |  |  |  |  |  |  |  |  |  |  |  |  |  |
|  | | cna | | | | | Total | | | |  |  |  |  |  |  |  |  |  |  |  |  |  |
|  |  | not found | | | found | |  |  |  |  |  |  |  |  |  |  |  |  |  |  |  |  |  |
| ica | not found | 165 | | | 9 | | 174 | | | |  |  |  |  |  |  |  |  |  |  |  |  |  |
|  | found | 22 | | | 4 | | 26 | | | |  |  |  |  |  |  |  |  |  |  |  |  |  |
| Total | | 187 | | | 13 | | 200 | | | |  |  |  |  |  |  |  |  |  |  |  |  |  |
| **Chi-Square Tests** | | | | | | | | | | | | | | | | | | | | |  |  |  |
|  | | | | Value | | df | | | | Asymp. Sig. (2-sided) | | | Exact Sig. (2-sided) | | | | Exact Sig. (1-sided) | | | |  |  |  |
| Pearson Chi-Square | | | | 3.882^a^ | | 1 | | | | .049 | | |  | | | |  | | | |  |  |  |
| Continuity Correction^b^ | | | | 2.383 | | 1 | | | | .123 | | |  | | | |  | | | |  |  |  |
| Likelihood Ratio | | | | 3.040 | | 1 | | | | .081 | | |  | | | |  | | | |  |  |  |
| Fisher's Exact Test | | | |  | |  | | | |  | | | .071 | | | | .071 | | | |  |  |  |
| Linear-by-Linear Association | | | | 3.862 | | 1 | | | | .049 | | |  | | | |  | | | |  |  |  |
| N of Valid Cases | | | | 200 | |  | | | |  | | |  | | | |  | | | |  |  |  |
| a. 1 cells (25.0%) have expected count less than 5. The minimum expected count is 1.69. | | | | | | | | | | | | | | | | | | | | |  |  |  |
| b. Computed only for a 2x2 table | | | | | | | | | | | | | | | | | | | | |  |  |  |
| **Directional Measures** | | | | | | | | | | | | | | | | | | | | | | | |
|  | | | | | | | | | | | | | | Value | | Asymp. Std. Error^a^ | | | Approx. T | Approx. Sig. | | |  |
| Nominal by Nominal | | | Lambda | | | | | | Symmetric | | | | | .000 | | .000 | | | .^b^ | .^b^ | | |  |
|  |  |  |  |  |  |  |  |  | ica Dependent | | | | | .000 | | .000 | | | .^b^ | .^b^ | | |  |
|  |  |  |  |  |  |  |  |  | cna Dependent | | | | | .000 | | .000 | | | .^b^ | .^b^ | | |  |
|  |  |  | Goodman and Kruskal tau | | | | | | ica Dependent | | | | | .019 | | .026 | | |  | .049^c^ | | |  |
|  |  |  |  |  |  |  |  |  | cna Dependent | | | | | .019 | | .026 | | |  | .049^c^ | | |  |
| a. Not assuming the null hypothesis. | | | | | | | | | | | | | | | | | | | | | | | |
| b. Cannot be computed because the asymptotic standard error equals zero. | | | | | | | | | | | | | | | | | | | | | | | |
| c. Based on chi-square approximation | | | | | | | | | | | | | | | | | | | | | | | |
| **Symmetric Measures** | | | | | | | | | | | | | | | | | | | | | |  |  |
|  | | | | | | | | Value | | | | Asymp. Std. Error^a^ | | | Approx. T^b^ | | | Approx. Sig. | | | |  |  |
| Nominal by Nominal | | | Phi | | | | | .139 | | | |  | | |  | | | .049 | | | |  |  |
|  |  |  | Cramer's V | | | | | .139 | | | |  | | |  | | | .049 | | | |  |  |
|  |  |  | Contingency Coefficient | | | | | .138 | | | |  | | |  | | | .049 | | | |  |  |
| Interval by Interval | | | Pearson's R | | | | | .139 | | | | .095 | | | 1.980 | | | .049^c^ | | | |  |  |
| Ordinal by Ordinal | | | Spearman Correlation | | | | | .139 | | | | .095 | | | 1.980 | | | .049^c^ | | | |  |  |
| N of Valid Cases | | | | | | | | 200 | | | |  | | |  | | |  | | | |  |  |
| a. Not assuming the null hypothesis. | | | | | | | | | | | | | | | | | | | | | |  |  |
| b. Using the asymptotic standard error assuming the null hypothesis. | | | | | | | | | | | | | | | | | | | | | |  |  |
| c. Based on normal approximation. | | | | | | | | | | | | | | | | | | | | | |  |  |

**ica * bap**

| **Crosstab** | | | | |
| --- | --- | --- | --- | --- |
| Count | | | | |
|  | | bap | | Total |
|  |  | not found | found |  |
| ica | not found | 173 | 1 | 174 |
|  | found | 26 | 0 | 26 |
| Total | | 199 | 1 | 200 |

| **Chi-Square Tests** | | | | | |
| --- | --- | --- | --- | --- | --- |
|  | Value | df | Asymp. Sig. (2-sided) | Exact Sig. (2-sided) | Exact Sig. (1-sided) |
| Pearson Chi-Square | .150^a^ | 1 | .698 |  |  |
| Continuity Correction^b^ | .000 | 1 | 1.000 |  |  |
| Likelihood Ratio | .279 | 1 | .597 |  |  |
| Fisher's Exact Test |  |  |  | 1.000 | .870 |
| Linear-by-Linear Association | .149 | 1 | .699 |  |  |
| N of Valid Cases | 200 |  |  |  |  |
| a. 2 cells (50.0%) have expected count less than 5. The minimum expected count is .13. | | | | | |
| b. Computed only for a 2x2 table | | | | | |

| **Directional Measures** | | | | | | | |
| --- | --- | --- | --- | --- | --- | --- | --- |
|  | | | Value | Asymp. Std. Error^a^ | Approx. T | Approx. Sig. |  |
| Nominal by Nominal | Lambda | Symmetric | .000 | .000 | .^b^ | .^b^ |  |
|  |  | ica Dependent | .000 | .000 | .^b^ | .^b^ |  |
|  |  | bap Dependent | .000 | .000 | .^b^ | .^b^ |  |
|  | Goodman and Kruskal tau | ica Dependent | .001 | .000 |  | .699^c^ |  |
|  |  | bap Dependent | .001 | .001 |  | .699^c^ |  |
| a. Not assuming the null hypothesis. | | | | | | | |
| b. Cannot be computed because the asymptotic standard error equals zero. | | | | | | | |
| c. Based on chi-square approximation | | | | | | | |

| **Symmetric Measures** | | | | | |
| --- | --- | --- | --- | --- | --- |
|  | | Value | Asymp. Std. Error^a^ | Approx. T^b^ | Approx. Sig. |
| Nominal by Nominal | Phi | -.027 |  |  | .698 |
|  | Cramer's V | .027 |  |  | .698 |
|  | Contingency Coefficient | .027 |  |  | .698 |
| Interval by Interval | Pearson's R | -.027 | .014 | -.386 | .700^c^ |
| Ordinal by Ordinal | Spearman Correlation | -.027 | .014 | -.386 | .700^c^ |
| N of Valid Cases | | 200 |  |  |  |
| a. Not assuming the null hypothesis. | | | | | |
| b. Using the asymptotic standard error assuming the null hypothesis. | | | | | |
| c. Based on normal approximation. | | | | | |

**Crosstabs**

| **Case Processing Summary** | | | | | | |
| --- | --- | --- | --- | --- | --- | --- |
|  | Cases | | | | | |
|  | Valid | | Missing | | Total | |
|  | N | Percent | N | Percent | N | Percent |
| fnbA * cna | 200 | 100.0% | 0 | 0.0% | 200 | 100.0% |
| fnbA * bap | 200 | 100.0% | 0 | 0.0% | 200 | 100.0% |
| fnbA * ica | 200 | 100.0% | 0 | 0.0% | 200 | 100.0% |

**fnbA * cna**

| **Crosstab** | | | | |
| --- | --- | --- | --- | --- |
| Count | | | | |
|  | | cna | | Total |
|  |  | not found | found |  |
| fnbA | not found | 164 | 7 | 171 |
|  | found | 23 | 6 | 29 |
| Total | | 187 | 13 | 200 |

| **Chi-Square Tests** | | | | | |
| --- | --- | --- | --- | --- | --- |
|  | Value | df | Asymp. Sig. (2-sided) | Exact Sig. (2-sided) | Exact Sig. (1-sided) |
| Pearson Chi-Square | 11.237^a^ | 1 | .001 |  |  |
| Continuity Correction^b^ | 8.672 | 1 | .003 |  |  |
| Likelihood Ratio | 8.184 | 1 | .004 |  |  |
| Fisher's Exact Test |  |  |  | .005 | .005 |
| Linear-by-Linear Association | 11.181 | 1 | .001 |  |  |
| N of Valid Cases | 200 |  |  |  |  |
| a. 1 cells (25.0%) have expected count less than 5. The minimum expected count is 1.89. | | | | | |
| b. Computed only for a 2x2 table | | | | | |

| **Directional Measures** | | | | | | |
| --- | --- | --- | --- | --- | --- | --- |
|  | | | Value | Asymp. Std. Error^a^ | Approx. T | Approx. Sig. |
| Nominal by Nominal | Lambda | Symmetric | .000 | .000 | .^b^ | .^b^ |
|  |  | fnbA Dependent | .000 | .000 | .^b^ | .^b^ |
|  |  | cna Dependent | .000 | .000 | .^b^ | .^b^ |
|  | Goodman and Kruskal tau | fnbA Dependent | .056 | .046 |  | .001^c^ |
|  |  | cna Dependent | .056 | .047 |  | .001^c^ |
| a. Not assuming the null hypothesis. | | | | | | |
| b. Cannot be computed because the asymptotic standard error equals zero. | | | | | | |
| c. Based on chi-square approximation | | | | | | |

| **Symmetric Measures** | | | | | |
| --- | --- | --- | --- | --- | --- |
|  | | Value | Asymp. Std. Error^a^ | Approx. T^b^ | Approx. Sig. |
| Nominal by Nominal | Phi | .237 |  |  | .001 |
|  | Cramer's V | .237 |  |  | .001 |
|  | Contingency Coefficient | .231 |  |  | .001 |
| Interval by Interval | Pearson's R | .237 | .100 | 3.433 | .001^c^ |
| Ordinal by Ordinal | Spearman Correlation | .237 | .100 | 3.433 | .001^c^ |
| N of Valid Cases | | 200 |  |  |  |
| a. Not assuming the null hypothesis. | | | | | |
| b. Using the asymptotic standard error assuming the null hypothesis. | | | | | |
| c. Based on normal approximation. | | | | | |

**fnbA * bap**

| **Crosstab** | | | | |
| --- | --- | --- | --- | --- |
| Count | | | | |
|  | | bap | | Total |
|  |  | not found | found |  |
| fnbA | not found | 170 | 1 | 171 |
|  | found | 29 | 0 | 29 |
| Total | | 199 | 1 | 200 |

| **Chi-Square Tests** | | | | | |
| --- | --- | --- | --- | --- | --- |
|  | Value | df | Asymp. Sig. (2-sided) | Exact Sig. (2-sided) | Exact Sig. (1-sided) |
| Pearson Chi-Square | .170^a^ | 1 | .680 |  |  |
| Continuity Correction^b^ | .000 | 1 | 1.000 |  |  |
| Likelihood Ratio | .314 | 1 | .575 |  |  |
| Fisher's Exact Test |  |  |  | 1.000 | .855 |
| Linear-by-Linear Association | .170 | 1 | .680 |  |  |
| N of Valid Cases | 200 |  |  |  |  |
| a. 2 cells (50.0%) have expected count less than 5. The minimum expected count is .15. | | | | | |
| b. Computed only for a 2x2 table | | | | | |

| **Directional Measures** | | | | | | | |
| --- | --- | --- | --- | --- | --- | --- | --- |
|  | | | Value | Asymp. Std. Error^a^ | Approx. T | Approx. Sig. |  |
| Nominal by Nominal | Lambda | Symmetric | .000 | .000 | .^b^ | .^b^ |  |
|  |  | fnbA Dependent | .000 | .000 | .^b^ | .^b^ |  |
|  |  | bap Dependent | .000 | .000 | .^b^ | .^b^ |  |
|  | Goodman and Kruskal tau | fnbA Dependent | .001 | .000 |  | .680^c^ |  |
|  |  | bap Dependent | .001 | .001 |  | .680^c^ |  |
| a. Not assuming the null hypothesis. | | | | | | | |
| b. Cannot be computed because the asymptotic standard error equals zero. | | | | | | | |
| c. Based on chi-square approximation | | | | | | | |

| **Symmetric Measures** | | | | | |
| --- | --- | --- | --- | --- | --- |
|  | | Value | Asymp. Std. Error^a^ | Approx. T^b^ | Approx. Sig. |
| Nominal by Nominal | Phi | -.029 |  |  | .680 |
|  | Cramer's V | .029 |  |  | .680 |
|  | Contingency Coefficient | .029 |  |  | .680 |
| Interval by Interval | Pearson's R | -.029 | .015 | -.411 | .682^c^ |
| Ordinal by Ordinal | Spearman Correlation | -.029 | .015 | -.411 | .682^c^ |
| N of Valid Cases | | 200 |  |  |  |
| a. Not assuming the null hypothesis. | | | | | |
| b. Using the asymptotic standard error assuming the null hypothesis. | | | | | |
| c. Based on normal approximation. | | | | | |

**fnbA * ica**

| **Crosstab** | | | | |
| --- | --- | --- | --- | --- |
| Count | | | | |
|  | | ica | | Total |
|  |  | not found | found |  |
| fnbA | not found | 158 | 13 | 171 |
|  | found | 16 | 13 | 29 |
| Total | | 174 | 26 | 200 |

| **Chi-Square Tests** | | | | | |
| --- | --- | --- | --- | --- | --- |
|  | Value | df | Asymp. Sig. (2-sided) | Exact Sig. (2-sided) | Exact Sig. (1-sided) |
| Pearson Chi-Square | 30.379^a^ | 1 | .000 |  |  |
| Continuity Correction^b^ | 27.177 | 1 | .000 |  |  |
| Likelihood Ratio | 22.683 | 1 | .000 |  |  |
| Fisher's Exact Test |  |  |  | .000 | .000 |
| Linear-by-Linear Association | 30.227 | 1 | .000 |  |  |
| N of Valid Cases | 200 |  |  |  |  |
| a. 1 cells (25.0%) have expected count less than 5. The minimum expected count is 3.77. | | | | | |
| b. Computed only for a 2x2 table | | | | | |

| **Directional Measures** | | | | | | |
| --- | --- | --- | --- | --- | --- | --- |
|  | | | Value | Asymp. Std. Error^a^ | Approx. T | Approx. Sig. |
| Nominal by Nominal | Lambda | Symmetric | .000 | .000 | .^b^ | .^b^ |
|  |  | fnbA Dependent | .000 | .000 | .^b^ | .^b^ |
|  |  | ica Dependent | .000 | .000 | .^b^ | .^b^ |
|  | Goodman and Kruskal tau | fnbA Dependent | .152 | .070 |  | .000^c^ |
|  |  | ica Dependent | .152 | .071 |  | .000^c^ |
| a. Not assuming the null hypothesis. | | | | | | |
| b. Cannot be computed because the asymptotic standard error equals zero. | | | | | | |
| c. Based on chi-square approximation | | | | | | |

| **Symmetric Measures** | | | | | |
| --- | --- | --- | --- | --- | --- |
|  | | Value | Asymp. Std. Error^a^ | Approx. T^b^ | Approx. Sig. |
| Nominal by Nominal | Phi | .390 |  |  | .000 |
|  | Cramer's V | .390 |  |  | .000 |
|  | Contingency Coefficient | .363 |  |  | .000 |
| Interval by Interval | Pearson's R | .390 | .092 | 5.955 | .000^c^ |
| Ordinal by Ordinal | Spearman Correlation | .390 | .092 | 5.955 | .000^c^ |
| N of Valid Cases | | 200 |  |  |  |
| a. Not assuming the null hypothesis. | | | | | |
| b. Using the asymptotic standard error assuming the null hypothesis. | | | | | |
| c. Based on normal approximation. | | | | | |

CROSSTABS

/TABLES=cna BY bap ica fnbA

/FORMAT=AVALUE TABLES

/STATISTICS=CHISQ CC PHI LAMBDA CORR

/CELLS=COUNT

/COUNT ROUND CELL.

**Crosstabs**

| **Case Processing Summary** | | | | | | |
| --- | --- | --- | --- | --- | --- | --- |
|  | Cases | | | | | |
|  | Valid | | Missing | | Total | |
|  | N | Percent | N | Percent | N | Percent |
| cna * bap | 200 | 100.0% | 0 | 0.0% | 200 | 100.0% |
| cna * ica | 200 | 100.0% | 0 | 0.0% | 200 | 100.0% |
| cna * fnbA | 200 | 100.0% | 0 | 0.0% | 200 | 100.0% |

**cna * bap**

| **Crosstab** | | | | |
| --- | --- | --- | --- | --- |
| Count | | | | |
|  | | bap | | Total |
|  |  | not found | found |  |
| cna | not found | 186 | 1 | 187 |
|  | found | 13 | 0 | 13 |
| Total | | 199 | 1 | 200 |

| **Chi-Square Tests** | | | | | |
| --- | --- | --- | --- | --- | --- |
|  | Value | df | Asymp. Sig. (2-sided) | Exact Sig. (2-sided) | Exact Sig. (1-sided) |
| Pearson Chi-Square | .070^a^ | 1 | .792 |  |  |
| Continuity Correction^b^ | .000 | 1 | 1.000 |  |  |
| Likelihood Ratio | .135 | 1 | .714 |  |  |
| Fisher's Exact Test |  |  |  | 1.000 | .935 |
| Linear-by-Linear Association | .070 | 1 | .792 |  |  |
| N of Valid Cases | 200 |  |  |  |  |
| a. 2 cells (50.0%) have expected count less than 5. The minimum expected count is .07. | | | | | |
| b. Computed only for a 2x2 table | | | | | |

| **Directional Measures** | | | | | | |
| --- | --- | --- | --- | --- | --- | --- |
|  | | | Value | Asymp. Std. Error^a^ | Approx. T | Approx. Sig. |
| Nominal by Nominal | Lambda | Symmetric | .000 | .000 | .^b^ | .^b^ |
|  |  | cna Dependent | .000 | .000 | .^b^ | .^b^ |
|  |  | bap Dependent | .000 | .000 | .^b^ | .^b^ |
|  | Goodman and Kruskal tau | cna Dependent | .000 | .000 |  | .792^c^ |
|  |  | bap Dependent | .000 | .000 |  | .792^c^ |
| a. Not assuming the null hypothesis. | | | | | | |
| b. Cannot be computed because the asymptotic standard error equals zero. | | | | | | |
| c. Based on chi-square approximation | | | | | | |

| **Symmetric Measures** | | | | | |
| --- | --- | --- | --- | --- | --- |
|  | | Value | Asymp. Std. Error^a^ | Approx. T^b^ | Approx. Sig. |
| Nominal by Nominal | Phi | -.019 |  |  | .792 |
|  | Cramer's V | .019 |  |  | .792 |
|  | Contingency Coefficient | .019 |  |  | .792 |
| Interval by Interval | Pearson's R | -.019 | .010 | -.263 | .793^c^ |
| Ordinal by Ordinal | Spearman Correlation | -.019 | .010 | -.263 | .793^c^ |
| N of Valid Cases | | 200 |  |  |  |
| a. Not assuming the null hypothesis. | | | | | |
| b. Using the asymptotic standard error assuming the null hypothesis. | | | | | |
| c. Based on normal approximation. | | | | | |

**cna * ica**

| **Crosstab** | | | | |
| --- | --- | --- | --- | --- |
| Count | | | | |
|  | | ica | | Total |
|  |  | not found | found |  |
| cna | not found | 165 | 22 | 187 |
|  | found | 9 | 4 | 13 |
| Total | | 174 | 26 | 200 |

| **Chi-Square Tests** | | | | | |
| --- | --- | --- | --- | --- | --- |
|  | Value | df | Asymp. Sig. (2-sided) | Exact Sig. (2-sided) | Exact Sig. (1-sided) |
| Pearson Chi-Square | 3.882^a^ | 1 | .049 |  |  |
| Continuity Correction^b^ | 2.383 | 1 | .123 |  |  |
| Likelihood Ratio | 3.040 | 1 | .081 |  |  |
| Fisher's Exact Test |  |  |  | .071 | .071 |
| Linear-by-Linear Association | 3.862 | 1 | .049 |  |  |
| N of Valid Cases | 200 |  |  |  |  |
| a. 1 cells (25.0%) have expected count less than 5. The minimum expected count is 1.69. | | | | | |
| b. Computed only for a 2x2 table | | | | | |

| **Directional Measures** | | | | | | |
| --- | --- | --- | --- | --- | --- | --- |
|  | | | Value | Asymp. Std. Error^a^ | Approx. T | Approx. Sig. |
| Nominal by Nominal | Lambda | Symmetric | .000 | .000 | .^b^ | .^b^ |
|  |  | cna Dependent | .000 | .000 | .^b^ | .^b^ |
|  |  | ica Dependent | .000 | .000 | .^b^ | .^b^ |
|  | Goodman and Kruskal tau | cna Dependent | .019 | .026 |  | .049^c^ |
|  |  | ica Dependent | .019 | .026 |  | .049^c^ |
| a. Not assuming the null hypothesis. | | | | | | |
| b. Cannot be computed because the asymptotic standard error equals zero. | | | | | | |
| c. Based on chi-square approximation | | | | | | |

| **Symmetric Measures** | | | | | |
| --- | --- | --- | --- | --- | --- |
|  | | Value | Asymp. Std. Error^a^ | Approx. T^b^ | Approx. Sig. |
| Nominal by Nominal | Phi | .139 |  |  | .049 |
|  | Cramer's V | .139 |  |  | .049 |
|  | Contingency Coefficient | .138 |  |  | .049 |
| Interval by Interval | Pearson's R | .139 | .095 | 1.980 | .049^c^ |
| Ordinal by Ordinal | Spearman Correlation | .139 | .095 | 1.980 | .049^c^ |
| N of Valid Cases | | 200 |  |  |  |
| a. Not assuming the null hypothesis. | | | | | |
| b. Using the asymptotic standard error assuming the null hypothesis. | | | | | |
| c. Based on normal approximation. | | | | | |

**cna * fnbA**

| **Crosstab** | | | | |
| --- | --- | --- | --- | --- |
| Count | | | | |
|  | | fnbA | | Total |
|  |  | not found | found |  |
| cna | not found | 164 | 23 | 187 |
|  | found | 7 | 6 | 13 |
| Total | | 171 | 29 | 200 |

| **Chi-Square Tests** | | | | | |
| --- | --- | --- | --- | --- | --- |
|  | Value | df | Asymp. Sig. (2-sided) | Exact Sig. (2-sided) | Exact Sig. (1-sided) |
| Pearson Chi-Square | 11.237^a^ | 1 | .001 |  |  |
| Continuity Correction^b^ | 8.672 | 1 | .003 |  |  |
| Likelihood Ratio | 8.184 | 1 | .004 |  |  |
| Fisher's Exact Test |  |  |  | .005 | .005 |
| Linear-by-Linear Association | 11.181 | 1 | .001 |  |  |
| N of Valid Cases | 200 |  |  |  |  |
| a. 1 cells (25.0%) have expected count less than 5. The minimum expected count is 1.89. | | | | | |
| b. Computed only for a 2x2 table | | | | | |

| **Directional Measures** | | | | | | | |
| --- | --- | --- | --- | --- | --- | --- | --- |
|  | | | Value | Asymp. Std. Error^a^ | Approx. T | Approx. Sig. |  |
| Nominal by Nominal | Lambda | Symmetric | .000 | .000 | .^b^ | .^b^ |  |
|  |  | cna Dependent | .000 | .000 | .^b^ | .^b^ |  |
|  |  | fnbA Dependent | .000 | .000 | .^b^ | .^b^ |  |
|  | Goodman and Kruskal tau | cna Dependent | .056 | .047 |  | .001^c^ |  |
|  |  | fnbA Dependent | .056 | .046 |  | .001^c^ |  |
| a. Not assuming the null hypothesis. | | | | | | | |
| b. Cannot be computed because the asymptotic standard error equals zero. | | | | | | | |
| c. Based on chi-square approximation | | | | | | | |

| **Symmetric Measures** | | | | | |
| --- | --- | --- | --- | --- | --- |
|  | | Value | Asymp. Std. Error^a^ | Approx. T^b^ | Approx. Sig. |
| Nominal by Nominal | Phi | .237 |  |  | .001 |
|  | Cramer's V | .237 |  |  | .001 |
|  | Contingency Coefficient | .231 |  |  | .001 |
| Interval by Interval | Pearson's R | .237 | .100 | 3.433 | .001^c^ |
| Ordinal by Ordinal | Spearman Correlation | .237 | .100 | 3.433 | .001^c^ |
| N of Valid Cases | | 200 |  |  |  |
| a. Not assuming the null hypothesis. | | | | | |
| b. Using the asymptotic standard error assuming the null hypothesis. | | | | | |
| c. Based on normal approximation. | | | | | |

**Crosstabs**

| **Case Processing Summary** | | | | | | |
| --- | --- | --- | --- | --- | --- | --- |
|  | Cases | | | | | |
|  | Valid | | Missing | | Total | |
|  | N | Percent | N | Percent | N | Percent |
| bap * ica | 200 | 100.0% | 0 | 0.0% | 200 | 100.0% |
| bap * fnbA | 200 | 100.0% | 0 | 0.0% | 200 | 100.0% |
| bap * cna | 200 | 100.0% | 0 | 0.0% | 200 | 100.0% |

**bap * ica**

| **Crosstab** | | | | |
| --- | --- | --- | --- | --- |
| Count | | | | |
|  | | ica | | Total |
|  |  | not found | found |  |
| bap | not found | 173 | 26 | 199 |
|  | found | 1 | 0 | 1 |
| Total | | 174 | 26 | 200 |

| **Chi-Square Tests** | | | | | |
| --- | --- | --- | --- | --- | --- |
|  | Value | df | Asymp. Sig. (2-sided) | Exact Sig. (2-sided) | Exact Sig. (1-sided) |
| Pearson Chi-Square | .150^a^ | 1 | .698 |  |  |
| Continuity Correction^b^ | .000 | 1 | 1.000 |  |  |
| Likelihood Ratio | .279 | 1 | .597 |  |  |
| Fisher's Exact Test |  |  |  | 1.000 | .870 |
| Linear-by-Linear Association | .149 | 1 | .699 |  |  |
| N of Valid Cases | 200 |  |  |  |  |
| a. 2 cells (50.0%) have expected count less than 5. The minimum expected count is .13. | | | | | |
| b. Computed only for a 2x2 table | | | | | |

| **Directional Measures** | | | | | | |
| --- | --- | --- | --- | --- | --- | --- |
|  | | | Value | Asymp. Std. Error^a^ | Approx. T | Approx. Sig. |
| Nominal by Nominal | Lambda | Symmetric | .000 | .000 | .^b^ | .^b^ |
|  |  | bap Dependent | .000 | .000 | .^b^ | .^b^ |
|  |  | ica Dependent | .000 | .000 | .^b^ | .^b^ |
|  | Goodman and Kruskal tau | bap Dependent | .001 | .001 |  | .699^c^ |
|  |  | ica Dependent | .001 | .000 |  | .699^c^ |
| a. Not assuming the null hypothesis. | | | | | | |
| b. Cannot be computed because the asymptotic standard error equals zero. | | | | | | |
| c. Based on chi-square approximation | | | | | | |

| **Symmetric Measures** | | | | | |
| --- | --- | --- | --- | --- | --- |
|  | | Value | Asymp. Std. Error^a^ | Approx. T^b^ | Approx. Sig. |
| Nominal by Nominal | Phi | -.027 |  |  | .698 |
|  | Cramer's V | .027 |  |  | .698 |
|  | Contingency Coefficient | .027 |  |  | .698 |
| Interval by Interval | Pearson's R | -.027 | .014 | -.386 | .700^c^ |
| Ordinal by Ordinal | Spearman Correlation | -.027 | .014 | -.386 | .700^c^ |
| N of Valid Cases | | 200 |  |  |  |
| a. Not assuming the null hypothesis. | | | | | |
| b. Using the asymptotic standard error assuming the null hypothesis. | | | | | |
| c. Based on normal approximation. | | | | | |

**bap * fnbA**

| **Crosstab** | | | | |
| --- | --- | --- | --- | --- |
| Count | | | | |
|  | | fnbA | | Total |
|  |  | not found | found |  |
| bap | not found | 170 | 29 | 199 |
|  | found | 1 | 0 | 1 |
| Total | | 171 | 29 | 200 |

| **Chi-Square Tests** | | | | | |
| --- | --- | --- | --- | --- | --- |
|  | Value | df | Asymp. Sig. (2-sided) | Exact Sig. (2-sided) | Exact Sig. (1-sided) |
| Pearson Chi-Square | .170^a^ | 1 | .680 |  |  |
| Continuity Correction^b^ | .000 | 1 | 1.000 |  |  |
| Likelihood Ratio | .314 | 1 | .575 |  |  |
| Fisher's Exact Test |  |  |  | 1.000 | .855 |
| Linear-by-Linear Association | .170 | 1 | .680 |  |  |
| N of Valid Cases | 200 |  |  |  |  |
| a. 2 cells (50.0%) have expected count less than 5. The minimum expected count is .15. | | | | | |
| b. Computed only for a 2x2 table | | | | | |

| **Directional Measures** | | | | | | |
| --- | --- | --- | --- | --- | --- | --- |
|  | | | Value | Asymp. Std. Error^a^ | Approx. T | Approx. Sig. |
| Nominal by Nominal | Lambda | Symmetric | .000 | .000 | .^b^ | .^b^ |
|  |  | bap Dependent | .000 | .000 | .^b^ | .^b^ |
|  |  | fnbA Dependent | .000 | .000 | .^b^ | .^b^ |
|  | Goodman and Kruskal tau | bap Dependent | .001 | .001 |  | .680^c^ |
|  |  | fnbA Dependent | .001 | .000 |  | .680^c^ |
| a. Not assuming the null hypothesis. | | | | | | |
| b. Cannot be computed because the asymptotic standard error equals zero. | | | | | | |
| c. Based on chi-square approximation | | | | | | |

| **Symmetric Measures** | | | | | |
| --- | --- | --- | --- | --- | --- |
|  | | Value | Asymp. Std. Error^a^ | Approx. T^b^ | Approx. Sig. |
| Nominal by Nominal | Phi | -.029 |  |  | .680 |
|  | Cramer's V | .029 |  |  | .680 |
|  | Contingency Coefficient | .029 |  |  | .680 |
| Interval by Interval | Pearson's R | -.029 | .015 | -.411 | .682^c^ |
| Ordinal by Ordinal | Spearman Correlation | -.029 | .015 | -.411 | .682^c^ |
| N of Valid Cases | | 200 |  |  |  |
| a. Not assuming the null hypothesis. | | | | | |
| b. Using the asymptotic standard error assuming the null hypothesis. | | | | | |
| c. Based on normal approximation. | | | | | |

**bap * cna**

| **Crosstab** | | | | |
| --- | --- | --- | --- | --- |
| Count | | | | |
|  | | cna | | Total |
|  |  | not found | found |  |
| bap | not found | 186 | 13 | 199 |
|  | found | 1 | 0 | 1 |
| Total | | 187 | 13 | 200 |

| **Chi-Square Tests** | | | | | |
| --- | --- | --- | --- | --- | --- |
|  | Value | df | Asymp. Sig. (2-sided) | Exact Sig. (2-sided) | Exact Sig. (1-sided) |
| Pearson Chi-Square | .070^a^ | 1 | .792 |  |  |
| Continuity Correction^b^ | .000 | 1 | 1.000 |  |  |
| Likelihood Ratio | .135 | 1 | .714 |  |  |
| Fisher's Exact Test |  |  |  | 1.000 | .935 |
| Linear-by-Linear Association | .070 | 1 | .792 |  |  |
| N of Valid Cases | 200 |  |  |  |  |
| a. 2 cells (50.0%) have expected count less than 5. The minimum expected count is .07. | | | | | |
| b. Computed only for a 2x2 table | | | | | |

| **Directional Measures** | | | | | | |
| --- | --- | --- | --- | --- | --- | --- |
|  | | | Value | Asymp. Std. Error^a^ | Approx. T | Approx. Sig. |
| Nominal by Nominal | Lambda | Symmetric | .000 | .000 | .^b^ | .^b^ |
|  |  | bap Dependent | .000 | .000 | .^b^ | .^b^ |
|  |  | cna Dependent | .000 | .000 | .^b^ | .^b^ |
|  | Goodman and Kruskal tau | bap Dependent | .000 | .000 |  | .792^c^ |
|  |  | cna Dependent | .000 | .000 |  | .792^c^ |
| a. Not assuming the null hypothesis. | | | | | | |
| b. Cannot be computed because the asymptotic standard error equals zero. | | | | | | |
| c. Based on chi-square approximation | | | | | | |

| **Symmetric Measures** | | | | | |
| --- | --- | --- | --- | --- | --- |
|  | | Value | Asymp. Std. Error^a^ | Approx. T^b^ | Approx. Sig. |
| Nominal by Nominal | Phi | -.019 |  |  | .792 |
|  | Cramer's V | .019 |  |  | .792 |
|  | Contingency Coefficient | .019 |  |  | .792 |
| Interval by Interval | Pearson's R | -.019 | .010 | -.263 | .793^c^ |
| Ordinal by Ordinal | Spearman Correlation | -.019 | .010 | -.263 | .793^c^ |
| N of Valid Cases | | 200 |  |  |  |
| a. Not assuming the null hypothesis. | | | | | |
| b. Using the asymptotic standard error assuming the null hypothesis. | | | | | |
| c. Based on normal approximation. | | | | | |
